# Hydrogen and Oxygen Mixture to Improve Cardiac Dysfunction and Myocardial Pathological Changes Induced by Intermittent Hypoxia in Rats

**DOI:** 10.1155/2019/7415212

**Published:** 2019-03-07

**Authors:** Ya-Shuo Zhao, Ji-Ren An, Shengchang Yang, Peng Guan, Fu-Yang Yu, Wenya Li, Jie-Ru Li, Yajing Guo, Zhi-Min Sun, En-Sheng Ji

**Affiliations:** ^1^Scientific Research Center, Hebei University of Chinese Medicine, Shijiazhuang 050200, China; ^2^Department of Physiology, Institute of Basic Medicine, Hebei University of Chinese Medicine, Shijiazhuang 050200, China

## Abstract

Obstructive sleep apnea (OSA) can cause intermittent changes in blood oxygen saturation, resulting in the generation of many reactive oxygen species (ROS). To discover new antioxidants and clarify the endoplasmic reticulum (ER) stress involved in cardiac injury in OSA, we established a chronic intermittent hypoxia (CIH) rat model with a fraction of inspired O_2_ (FiO_2_) ranging from 21% to 9%, 20 times/h for 8 h/day, and the rats were treated with H_2_-O_2_ mixture (67% hydrogen and 33% oxygen) for 2 h/day for 35 days. Our results showed that H_2_-O_2_ mixture remarkably improved cardiac dysfunction and myocardial fibrosis. We found that H_2_-O_2_ mixture inhalation declined ER stress-induced apoptosis via three major response pathways: PERK-eIF2*α*-ATF4, IRE 1-XBP1, and ATF 6. Furthermore, we revealed that H_2_-O_2_ mixture blocked c-Jun N-terminal kinase- (JNK-) MAPK activation, increased the ratio of Bcl-2/Bax, and inhibited caspase 3 cleavage to protect against CIH-induced cardiac apoptosis. In addition, H_2_-O_2_ mixture considerably decreased ROS levels via upregulating superoxide dismutase (SOD) and glutathione (GSH) as well as downregulating NADPH oxidase (NOX 2) expression in the hearts of CIH rats. All the results demonstrated that H_2_-O_2_ mixture significantly reduced ER stress and apoptosis and that H_2_ might be an efficient antioxidant against the oxidative stress injury induced by CIH.

## 1. Introduction

Obstructive sleep apnea (OSA) is a common breathing disorder and characterized by recurrent episodes of upper airway obstruction during sleep [1]. Clinical data have shown that the incidence of OSA was approximately 15-24% in adults [2] and that OSA was accompanied by multiple cardiovascular disorders, such as hypertension, heart failure, and atherosclerosis [3, 4]. OSA patients showed long-term arterial oxygen saturation fluctuations and frequent sleep apnea, exposing them to a specific internal environment with chronic intermittent hypoxia (CIH) and recurrent hypoxia [5–7].

Sun et al. found that increases in the left ventricular diameter and ventricular mass in OSA patients correlated with the severity of the disease [8]. Clinically, continuous positive airway pressure (CPAP) is the most widely used treatment for OSA during sleep [9]. Unfortunately, CPAP lacks stability and is not effective in reducing the cardiac damage caused by OSA. How can the cardiac damage caused by CIH be effectively reduced? It is necessary to further study the molecular mechanism of injury that is induced by CIH and seek a more effective treatment method for CIH.

The endoplasmic reticulum (ER) is a crucial organelle for protein synthesis, folding, and secretion. When cells are stimulated by ischemia, hypoxia, or oxidative stress, unfolded protein and incorrect proteins accumulate in the ER, triggering the unfolded protein response (UPR), which is called ER stress [10, 11]. UPR activation is regulated by a molecular chaperone protein 78 KD glucose-regulated protein named Bip/GRP 78 [10, 12]. During ER stress, Bip and GRP 78 are separated first, and protein kinase-like kinase (PERK), inositol-requiring enzyme 1 (IRE 1), and transcription factor 6 (ATF 6) are activated [10, 11]. However, prolonged or severe ER stress could induce cell apoptosis [13, 14]. The apoptosis caused by ER stress is stimulated through the proapoptotic transcriptional factor C/EBP homologous protein (CHOP) [15]. Activated ATF 6, PERK, and IRE 1 accelerate the activation of the CHOP protein and lead to cell apoptosis [13].

During the process of low oxygen/reoxygenation induced by CIH, a large number of reactive oxygen species (ROS) are generated and trigger oxidative stress damage [12, 14, 16, 17]. Xu et al. found that the ER structure was changed and that the GRP 78, CHOP, and caspase 12 levels were increased in the hippocampus of adult mice exposed to CIH for 21 d [12]. These results suggested that the ER stress response was an early event in cardiac apoptosis caused by CIH [18]. Cai et al. revealed that the PERK-eIF2*α*-ATF 4 signaling pathway was involved in apoptosis in growing rats when they were exposed to long-term CIH [19]. The study showed that IRE 1-XBP 1 and ATF 6 expression was dramatically increased in rat cardiac tissues when exposed to CIH for 5 weeks [20]. Tauroursodeoxycholic acid (TUDCA), an ER stress inhibitor, could have inhibited ER stress activation and apoptosis in the hippocampus of the rat CIH model [12, 19]. TUDCA also attenuated the activation of PERK, IRE 1, and ATF 6 in the liver of a mouse CIH model [21]. Therefore, the inhibition of ER stress might be an effective way to reduce cardiac injury when animals are exposed to CIH.

As a “novel” antioxidant, H_2_ has received extensive attention and is widely used in the prevention and treatment of various diseases [22, 23]. It has been confirmed that H_2_ is very stable and easily penetrates cell membranes and barriers without affecting basic metabolism in cells [24]. A study has shown that H_2_-rich saline could have weakened hippocampal ER stress after cardiac arrest in a rat model [25]. H_2_-rich saline was also efficiently used to attenuate the permeability of the blood-brain barrier and microvascular endothelial cell apoptosis from cardiopulmonary bypass in a rat model [26]. H_2_ inhibited isoproterenol-induced cardiac hypertrophy by blocking excess ROS and mitochondrial damage [27].

Our previous research showed that H_2_ inhalation significantly increased the level of total superoxide dismutase (T-SOD) in the serum of a CIH rat model [28]. Whether H_2_ can attenuate cardiac ER stress and apoptosis remains unclear. To better understand the cardioprotective mechanism of H_2_, we investigated the effect of H_2_ on cardiac ER stress and apoptosis in a rat model exposed to CIH.

## 2. Materials and Methods

### 2.1. Experimental Animals and the CIH Model

All procedures were carried out in accordance with the National Institutes of Health Guide for the Care and Use of Laboratory Animals and were approved by the Animal Care and Use Committee of Medical Ethics of Hebei University of Chinese Medicine (no. HUCM-20117-010). Adult male Sprague-Dawley rats (190-220 g) were purchased from Beijing Vital River Laboratory Animal Technology Co. Ltd. (Beijing, China). All rats were housed under a constant temperature (22 ± 2°C) and controlled illumination (12 h light and 12 h dark cycle) and given free access to food and water. All rats were allowed to adapt to their living conditions for at least 7 days before the experiment.

The SD rats (*n* = 36) were randomly divided into four groups (*n* = 9 for each group): normoxia control group (normoxia), normoxia H_2_-O_2_ mixture-treated group (H_2_), CIH model group (CIH), and H_2_-O_2_ mixture-treated CIH model group (CIH+H_2_). During the experiment, all rats were housed in chambers with a controlled gas delivery system. The fraction of inspired oxygen (FiO_2_) provided to the chambers for the CIH and the CIH+H_2_ groups declined from 21% to 9% within 90 s and then gradually increased to 21% via reoxygenation within 90 s. The exposure cycle was repeated every 3 min from 8:00 to 16:00 everyday for 35 days. The rats in the normoxia and H_2_ groups received air containing 21% O_2_. In addition, the rats in the CIH+H_2_ and H_2_ groups were successively given H_2_-O_2_ mixture gas from 17:00 to 19:00 everyday for 35 days. The H_2_-O_2_ mixture gas was obtained from water electrolyzation with a hydrogen oxygen nebulizer (AMS-H-01, Asclepius Meditec, Shanghai, China) and consisted of 67% H_2_ and 33% O_2_. During the experiment, the rats were placed in a transparent chamber, and the mixed gas went through the chamber at a rate of 200 ml/min. The concentration of mixed gas was monitored by a detector (Thermo Fisher, MA, USA).

### 2.2. Echocardiography

Echocardiographic analysis was performed by a high-resolution ultrasound imaging system (Vevo 2100, VisualSonics Inc., Toronto, Canada) with an MS-250 probe. First, the rat was anesthetized with 2.5% isoflurane in 95% oxygen and 5% carbon dioxide, and the hair was removed with depilatory cream. The QRS and T waves were used as indicators of the systolic and diastolic phases, and the left ventricular diameter was measured by combining the opening and closing of the mitral valve on the image. M-mode recordings detected the left ventricular end-diastolic diameter (LVEDd) and left ventricular end-systolic diameter (LVEDs). The left ventricular end-systolic volume (LVESV) = 7/(2.4 + LVEDs) × LVEDs^3^ × 1000, left ventricular end-diastolic volume (LVEDV) = 7/(2.4 + LVEDd) × LVEDd^3^ × 1000, and ejection fraction (EF) = (LVEDV–LVESV)/LVEDV × 100% were also measured. Four-chamber echocardiography showed the maximum flow rate in the early diastole (E), maximum flow rate in the systolic phase (A) of the mitral valve (MV), isovolumic contraction period (IVCT), isovolumic relaxation phase (IVRT), and ejection period (ET). The value of the ratio of MV E/A and Tei index = (IVCT + IVRT)/ET was used as indicators to reflect the changes in cardiac function. The technical parameters of the echocardiograph were the same for all test objects, and the average values were taken for at least 3 continuous cycles. The echocardiographic measurements were taken by a blinded observer.

### 2.3. Histological Assessment

The hearts were removed, soaked in 4% polyformaldehyde, washed with tap water, and dehydrated with serial dilutions of alcohol. The heart tissues were transparent in xylene and embedded in paraffin for 24 h. The paraffin-enclosed tissue was sliced into 5 *μ*m sections by a sliding microtome (CM1950, Leica, Solms, Germany). The sections were dewaxed by xylene and rehydrated by a sequence of 100% to 70% ethanol. Hematoxylin and eosin (H&E) staining was used to detect changes in the basic tissue and structure of the heart. Sections were continuously stained with hematoxylin, differentiated with eosin, and dehydrated. Masson's trichrome (MT) staining was used to identify the collagenous fibrous area of the heart. The sections were stained with Masson's trichrome stain, distilled water, phosphomolybdic, and aniline blue solution and then differentiated in order. Finally, the sections were dehydrated, mounted, and imaged using an electric light microscope (DM3000, Leica, Solms, Germany). Image-Pro Plus 6.0 image analysis software was used to analyze and calculate the myocardial collagen volume fraction = collagen area/the total myocardial area (100%).

### 2.4. Measurement of Oxidative Stress

T-SOD and glutathione (GSH) were the antioxidant indices, while malonyldialdehyde (MDA) was a lipid peroxide marker. The activities of T-SOD and GSH were measured with the hydroxylamine method, and MDA was measured using the thiobarbituric acid method as previously described [29]. First, the left ventricle tissues were prepared to obtain a 10% (*w*/*v*) ice-buffered homogenate. After centrifugation at 2500 rpm for 10 min (4°C), the supernatant was collected to detect the protein content with a BCA kit (CW0014S, Cwbiotech, Beijing, China). The measurements were all performed according to the manufacturer's instructions (Nanjing Jiancheng Bioengineering Institute, Nanjing, China). The T-SOD, GSH, and MDA levels were measured with a multimode microplate reader (Varioskan LUX, Thermo Fisher, MA, USA) at wavelengths of 550 nm, 532 nm, and 550 nm, respectively.

### 2.5. Detection of Apoptosis

Apoptosis in the heart tissue was detected by the terminal deoxynucleotidyl transferase-mediated FITC-dUDP nick-end labeling (TUNEL) method. Heart tissue sections were dewaxed and incubated with 3% H_2_O_2_ for 20 min at room temperature. The reaction mixture (TUN11684817, Roche, Basel, Switzerland) was dropped onto slides and incubated at 37°C for 60 min. After the sections were rinsed 3 times, they were incubated in DAPI (2 mg/ml, Solarbio, Beijing, China) for 5 min. Finally, the number of TUNEL-positive/DAPI-stained apoptotic bodies was counted with an electric light microscope (DM3000, Leica, Solms, Germany).

### 2.6. Western Blotting

The cardiac tissues were homogenized in RIPA lysis buffer with a proteinase inhibitor. The suspension was centrifuged at 12,000 g for 20 min at 4°C, the supernatant was collected, and the protein concentration was measured with a BCA protein assay kit (CW0014S, Cwbiotech, Beijing, China). Thirty micrograms of proteins was separated by SDS-PAGE and transferred onto polyvinylidene fluoride membranes. After blocking with 5% nonfat milk, the blots were incubated with primary antibodies against CHOP (GTX32616, GeneTex, Irvine, USA), GRP 78 (ARG20531, Arigo Biolaboratories, Taiwan, China), caspase 12 (ARG55177, Arigo Biolaboratories, Taiwan, China), p-PERK (DF7576, Affinity Biosciences, OH, USA), PERK (AF5304, Affinity Biosciences, OH, USA), p-eIF 2*α* (AF3087, Affinity Biosciences, OH, USA), eIF2*α* (A0764, ABclonal Biotechnology, Boston, USA), p-IRE 1 (AF7150, Affinity Biosciences, OH, USA), IRE 1 (DF7709, Affinity Biosciences, OH, USA), ATF 4 (Ab1371, Abcam, Cambridge, UK), ATF 6 (A2570, ABclonal Biotechnology, Boston, USA), XBP 1 (AF5110, Affinity Biosciences, OH, USA), caspase 3 (9665, Cell Signaling Technology, Danvers, USA), p-JNK (4671, Cell Signaling Technology, Danvers, USA), JNK (ARG51218, Arigo Biolaboratories, Taiwan, China), Bcl-2 (YT0470, Immunoway, Plano, USA), Bax (GB11007, Servicebio, Wuhan, China), NOX 2 (GTX56278, GeneTex, Irvine, USA), and *β*-tubulin (GB13017-2, Servicebio, Wuhan, China) overnight at 4°C. The blots were washed with TBST and then incubated with the secondary antibody conjugated with horseradish peroxide (Biosharp, Hefei, China) for 90 min at room temperature. The chemiluminescence method (CW0049S, Cwbiotech, Beijing, China) was used to detect the immunoreactive proteins with a multifunctional laser scanning system (Fusion FX5 Spectra, Vilber, Paris, France). All the analyses were repeated at least three times. The densities of the positive proteins were quantified by Image J and expressed as a ratio to *β*-tubulin.

### 2.7. Statistical Analyses

The results are presented as the mean ± SEM. The statistical analysis was carried out using a two-way ANOVA followed by Tukey's post hoc test. The significance level was *p* < 0.05.

## 3. Results

### 3.1. H_2_-O_2_ Mixture Remarkably Improved Cardiac Dysfunction

**Figure 1:**
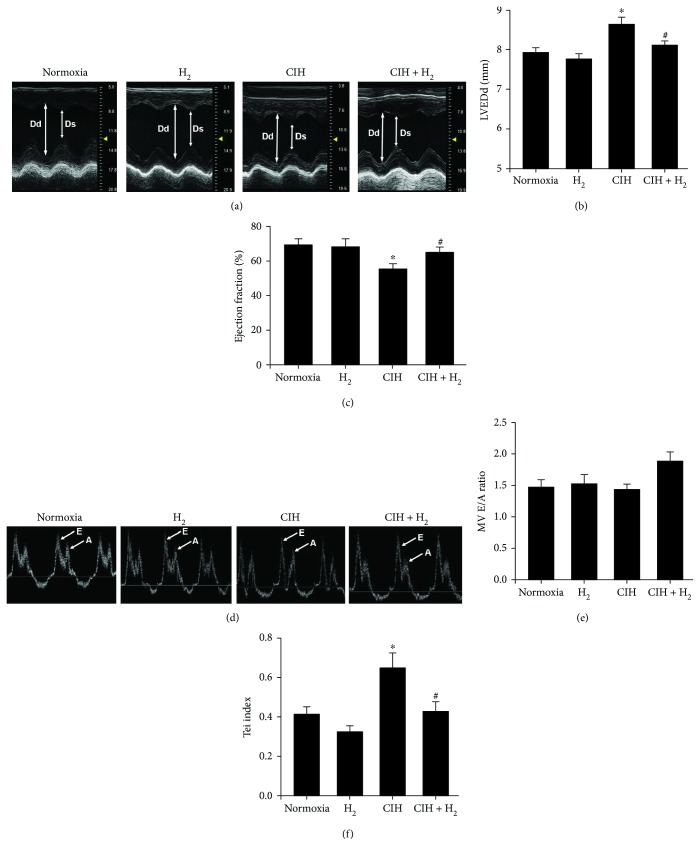
The effect of H_2_-O_2_ mixture on improving cardiac dysfunction in a rat model when exposed to CIH for 35 d. (a) M-model echocardiography of the short axial section of the thoracic bones in rats. Dd: end-diastolic diameter of the left ventricle; Ds: end-systolic diameter of the left ventricle. (b) The mean value of the left ventricular end-diastolic internal diameter (LVEDd). (c) The ejection fraction (EF) of the left ventricle. (d) Rat four-chamber echocardiograph with atrial contraction waves. (e) The velocity ratio of the E peak to the A peak in the cardiac mitral valve (MV E/A); (f) Tei index = (IVCT + IVRT)/ET. ^∗^*p* < 0.05 vs. normoxia group; ^#^*p* < 0.05 vs. CIH group; *n* = 5.

Echocardiography was utilized to detect the rat cardiac systolic and diastolic functions. M-mode recordings showed higher values of LVEDd (Figures 1(a) and 1(b)) and lower EF (Figure 1(c)), indicating cardiac systolic dysfunction in the CIH rat model. However, the groups with the H_2_-O_2_ mixture treatment showed lower LVEDd values and higher EF than did the CIH group (Figures 1(a)–1(c)). Four-chamber echocardiography was used to evaluate the cardiac diastolic function (Figure 1(d)). The ratio of MV E/A showed no significant difference among the four groups (Figure 1(e)). CIH rats exhibited high values of the Tei index, indicating that their cardiac diastolic function was impaired (Figure 1(f)). However, H_2_-O_2_ mixture treatment decreased the Tei value to the normal level and improved cardiac diastolic function induced by CIH (Figure 1(f)). These results suggested that H_2_-O_2_ mixture treatment was an effective way to reduce cardiac systolic and diastolic dysfunctions in rats when exposed to CIH.

### 3.2. H_2_-O_2_ Mixture Significantly Reduced Cardiac Histological Changes

**Figure 2:**
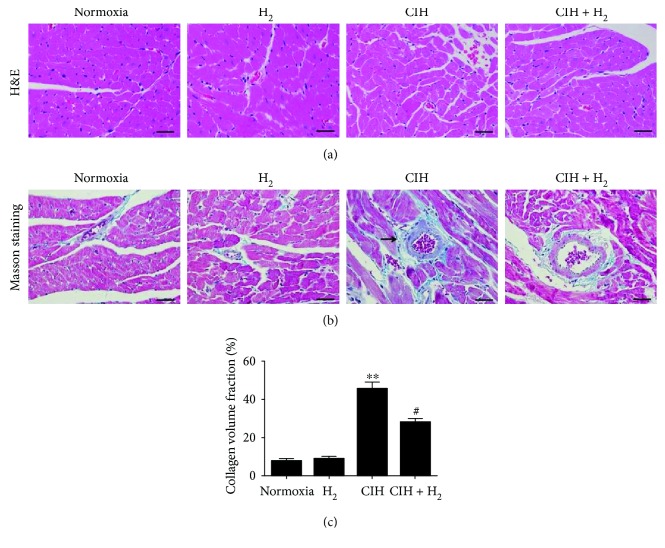
The histopathologic changes from H_2_-O_2_ mixture treatment during CIH for 35 d. (a) H&E staining showed the cardiac architecture in the left ventricle of the four different groups (scale bar = 25 *μ*m); (b) Masson's trichrome staining showed the collagen deposition in the left ventricle of the heart (scale bar = 25 *μ*m); (c) the myocardial collagen volume fractions were counted as shown by Masson's trichrome staining. ^∗^*p* < 0.05 vs. normoxia group; ^#^*p* < 0.05 vs. CIH group; *n* = 3.

Did H_2_-O_2_ mixture provide protection against pathological changes in the heart of the CIH rat model? H&E and MT staining were used to analyze the left ventricle of the rats. H&E staining showed a clear and complete cardiomyocyte structure and endocardium in normal rats (Figure 2(a)). H_2_-O_2_ mixture improved the widespread myocardial structural disorder in the CIH group (Figure 2(a)). In addition, MT staining was used to evaluate myocardial fibrosis in the left ventricle. As shown in Figures 2(b) and 2(c), collagen accumulation shown in blue was increased in the CIH group. However, the groups treated with H_2_-O_2_ mixture had a significantly lower collagen volume fraction in the left ventricle of the heart than did the CIH group. Altogether, H_2_-O_2_ mixture improved myocardial structure disorder and collagen deposition when rats were exposed to CIH.

### 3.3. H_2_-O_2_ Mixture Remarkably Attenuated CIH-Induced ER Stress

**Figure 3:**
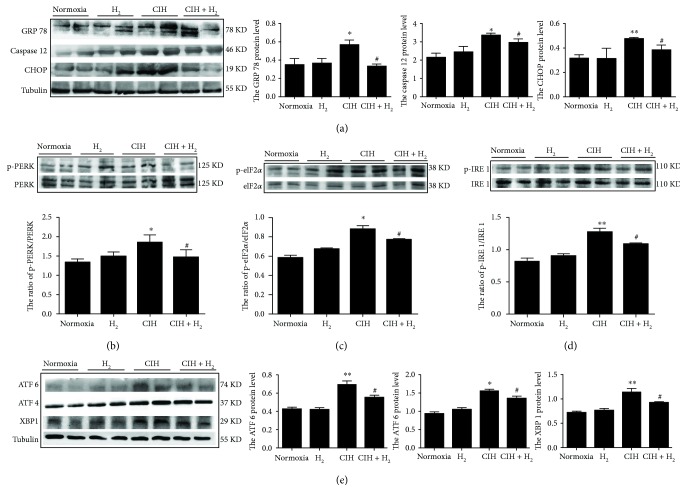
The H_2_-O_2_ mixture-induced inhibition of ER stress caused by CIH for 35 d: (a) the ER stress markers GRP 78, caspase 12, and CHOP protein expressions; (b–d) the ratios of p-PERK, p-eIF2*α*/eIF2*α*, and p-IRE 1/IRE 1 in the left ventricle; (e) ATF 6, ATF 4, and XBP 1 protein expressions. The results are presented as the mean ± SEM. ^∗^*p* < 0.05; ^∗∗^*p* < 0.01 vs. normoxia group; ^#^*p* < 0.05 vs. CIH group; *n* = 3.

To further study the protective mechanism of H_2_-O_2_ mixture against cardiac injury induced by CIH, we first evaluated the expression of ER stress markers. GRP 78, CHOP, and caspase 12 were all increased in the cardiac tissue when exposed to CIH (Figure 3(a)). Then, we examined the three major pathways involved in ER stress-induced apoptosis. In the CIH group, the protein levels of p-PERK, p-eIF2*α*, and ATF 4 significantly increased compared to those in the normoxia group (Figures 3(b), 3(c), and 3(e)). We found that p-IRE 1, XBP 1, and ATF 6 were also elevated after CIH exposure (Figures 3(d) and 3(e)). However, the activation of p-PERK, p-eIF2*α*, and p-IRE 1 was inhibited in the H_2_ group compared to that in the CIH group (Figures 3(b)–3(d)). Our results revealed that the protein levels of ATF 4, ATF 6, and XBP 1 (Figure 3(e)) were all decreased when the CIH rat model was treated with H_2_-O_2_ mixture. These results suggested that H_2_-O_2_ mixture could reduce ER stress-induced apoptosis via the PERK-eIF2*α*-ATF 4, IRE 1-XBP 1, and ATF 6 pathways.

### 3.4. H_2_-O_2_ Mixture Greatly Inhibited JNK-MAPK-Induced Apoptosis

**Figure 4:**
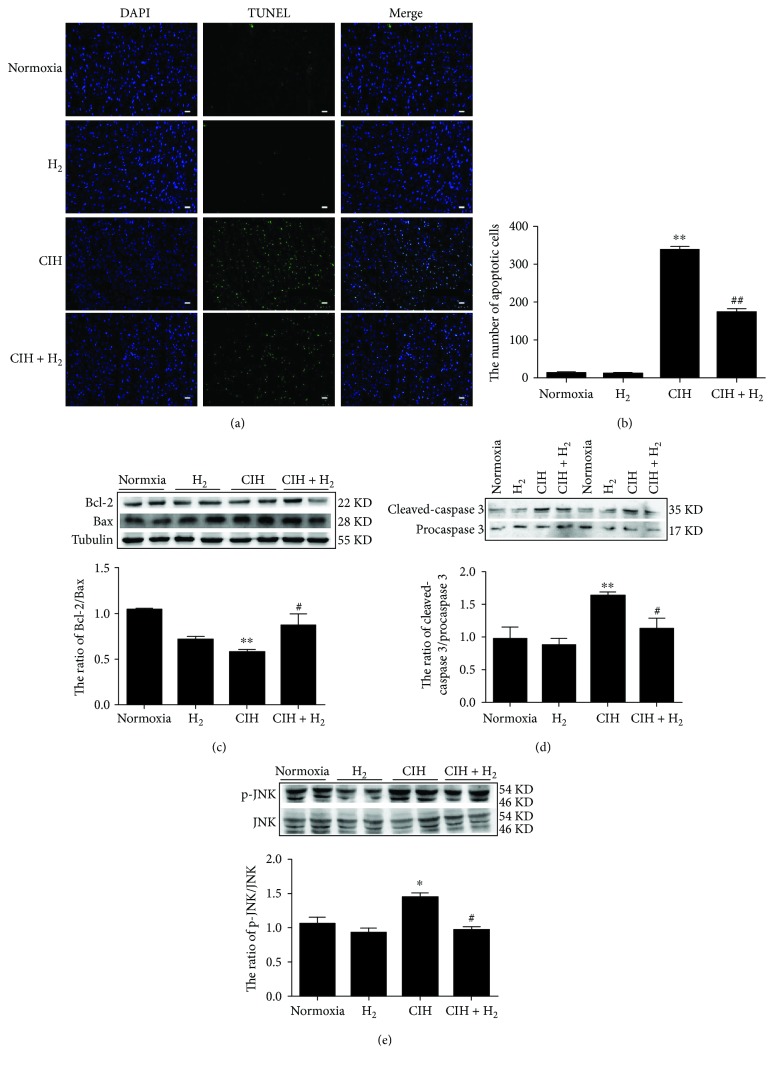
The effect of the H_2_-O_2_ mixture treatment on cardiomyocyte apoptosis in the CIH model: (a) TUNEL staining (scale bar = 25 *μ*m); (b) the number of apoptotic bodies as shown in (a); (c) the ratio of Bcl-2/Bax; (d) the ratio of cleaved-caspase 3/procaspase 3; (e) the ratio of p-JNK and JNK. The results are presented as the mean ± SEM. ^∗^*p* < 0.05; ^∗∗^*p* < 0.01 vs. normoxia group; ^#^*p* < 0.05 vs. CIH group; *n* = 3.

Did H_2_-O_2_ mixture protect against myocardial cell apoptosis via the mitochondrial pathway? First, we detected the occurrence of cardiac apoptosis when rats were exposed to CIH. As shown in Figure 4(a), a large number of apoptotic bodies were observed in the left ventricle when the rat model was exposed to CIH. The total number of apoptotic bodies in the CIH+H_2_ group was strikingly lower than that in the CIH group (Figures 4(a) and 4(b)). At the same time, we detected the effect of H_2_-O_2_ mixture on some apoptotic signaling molecules. Our results indicated that H_2_-O_2_ mixture significantly increased the decrease in Bcl-2 and reduced the increase in Bax that were induced by CIH (Figure 4(c)). Similarly, the ratio of cleaved-caspase 3/procaspase 3 in the left ventricle was increased in the CIH+H_2_ group compared to that in the CIH group (Figure 4(d)). Furthermore, we found that the c-Jun N-terminal kinase- (JNK-) MAPK pathway was activated in the left ventricle when the rats were exposed to CIH (Figure 4(e)). However, H_2_-O_2_ mixture markedly suppressed the phosphorylation of JNK (Figure 4(e)) in the left ventricle. The results indicated that H_2_-O_2_ mixture could attenuate myocardial cell apoptosis via the mitochondrial pathway induced by CIH.

### 3.5. H_2_-O_2_ Mixture Efficiently Reduced Oxidative Stress in Cardiac Tissue

**Figure 5:**
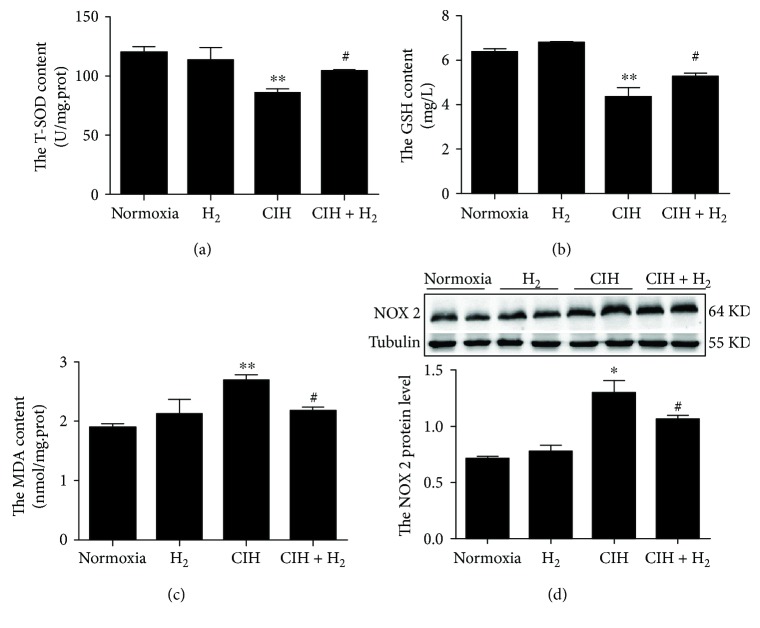
The effect of H_2_-O_2_ mixture on CIH-induced oxidative stress in the heart: (a, b) total superoxide dismutase (T-SOD) and glutathione (GSH) activities; (c) the content of malondialdehyde (MDA); (d) the NOX 2 protein level. The results are presented as the mean ± SEM. ^∗^*p* < 0.05 vs. normoxia group; ^#^*p* < 0.05 vs. CIH group; *n* = 3.

Oxidative stress might be an inducer of cardiac apoptosis when animals are exposed to CIH. We investigated whether H_2_-O_2_ mixture enhanced the antioxidant capacity to protect against CIH-induced oxidative stress injury. SOD and GSH are essentially endogenous antioxidants that scavenge superoxide anion radicals and hydrogen peroxide [30]. T-SOD and GSH activities were substantially elevated in the CIH+H_2_ group compared to the CIH group (Figures 5(a) and 5(b)). However, the MDA content declined when the rat model was treated with H_2_-O_2_ mixture during CIH (Figure 5(c)). NADPH oxidase is an important source of ROS under some pathological conditions [29]. We found that the protein level of NOX 2, which is an important subtype of NADPH oxidase, was decreased when the rats were treated with H_2_-O_2_ mixture compared to that in the CIH group (Figure 5(d)). The results implied that H_2_-O_2_ mixture had ability to scavenge ROS in cardiac tissue exposed to CIH.

## 4. Discussion

OSA leads to CIH and contributes to cardiovascular diseases [31]. In this study, echocardiography revealed that cardiac systolic function declined, as shown by higher values of LVEDd and lower EF in CIH rats than in normoxia rats, which is consistent with clinical findings [8]. The morphological results showed that the CIH rats showed myocardial fiber fractures and disorders, which might be an important cause of fibrosis in the heart [32]. In our study, we found that H_2_-O_2_ mixture inhalation could protect against the cardiac dysfunction and structural disorders induced by CIH in vivo. Furthermore, our study demonstrated that the cardioprotective effect of H_2_-O_2_ mixture was due to decreased ROS accumulation by reducing NADPH oxidase expression and blocking the PERK-eIF2*α*-ATF4, IRE-XBP1, ATF 6, and JNK signaling that is involved in ER stress and apoptosis in CIH rats. Similar to other studies [33, 34], there was no significant difference between the normoxia and H_2_-O_2_ mixture-treated rats. Therefore, we think that H_2_ plays the protective effect against CIH-induced cardiac damage.

A study showed that CHOP levels were significantly increased in many cardiac-related diseases [15], and another study showed that a deficiency in the CHOP gene reduced apoptosis in response to ER stress [35]. Our results showed that CHOP proteins were significantly increased in the left ventricle of the CIH rat model, indicating that the heart was undergoing apoptosis. During CIH, a large number of ROS are generated [11, 12], which further accelerates the separation of GRP 78 from Bip [20] and activates PERK, IRE 1, and ATF 6 [36, 37]. The activation of PERK, IRE 1, and ATF 6 is all involved in apoptosis via CHOP [15, 38]. Activated PERK can phosphorylate eIF2*α* at Ser 51, which selectively induces the translation and protein synthesis of ATF 4 [39]. ATF 4 is a transcription factor and enhances CHOP translation [38]. Additionally, XBP 1 is spliced by the endoribonuclease of IRE 1 under ER stress [40] and becomes a potent transcription factor for CHOP [38]. Our results showed that the PERK-eIF2*α*-ATF4, IRE 1-XBP1, and ATF 6 pathways were all inhibited in the CIH+H_2_ group compared to the CIH group (Figure 3). These results suggested that ER stress-induced apoptosis was inhibited in cardiac tissues when CIH rats inhaled H_2_-O_2_ mixture.

**Figure 6:**
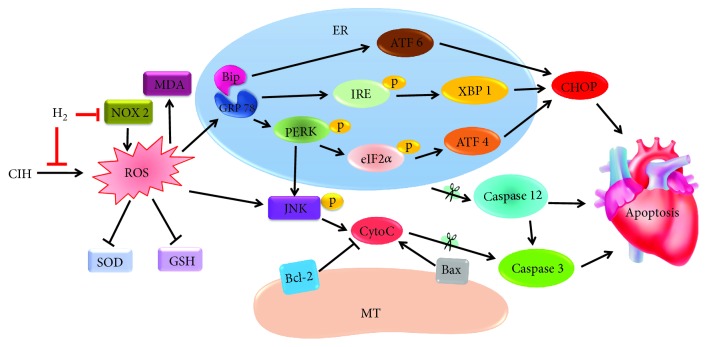
A schematic graph of the proposed cardioprotective mechanism of H_2_ when rats were exposed to CIH. H_2_ reduced the high level of ROS by elevating SOD and GSH activities and decreasing NOX 2 and the MDA content. H_2_ inhibited ER stress by downregulating GRP 78, CHOP, and caspase 12 proteins. H_2_ decreased CIH-induced apoptosis via three major ER stress response pathways: PERK-eIF2*α*-ATF4, IRE 1-XBP1, and ATF 6. H_2_ attenuated the JNK-MAPK pathway involved in apoptosis.

Previous studies confirmed that activated JNK-MAPK was involved in cell apoptosis induced by oxidative stress [29, 41]. The high level of ROS directly accelerates JNK-MAPK signaling activation, resulting in apoptosis [20, 29]. Activated JNK promotes Bax translocation from the cytoplasm to the mitochondria and decreases the expression of the antiapoptotic factor Bcl-2, resulting in the release of cytochrome C (Cyto C) into the cytoplasm [42]. Dysfunction in mitochondria would activate caspase 3, degrade the downstream substrate, and eventually lead to apoptosis [43]. Our results showed a lower ratio of Bcl-2/Bax, and the activation of caspase 3 and JNK was induced during CIH (Figure 4). We found that the JNK-MAPK pathway was significantly inhibited when CIH rats were treated with H_2_-O_2_ mixture. Furthermore, studies have reported that JNK-MAPK signaling was also related to ER-induced apoptosis [20, 44]. Studies revealed that activated PERK could induce JNK phosphorylation [11] and that phosphorylated IRE 1 was able to recruit TNFR-associated factor-2 (TRAF-2) and activate the downstream target phospho-JNK-MAPK [44]. In addition, activated CHOP is also involved in apoptosis via downregulating Bcl-2 expression [45]. Our results showed that p-PERK, p-IRE, and CHOP were all inhibited when rats were treated with H_2_-O_2_ mixture (Figure 3). Therefore, JNK-MAPK signaling played multiple roles in the cardioprotective effects of H_2_-O_2_ mixture (Figure 6).

During the hypoxia/reoxygenation process, ER stress causes calcium ions to continuously drain from the ER and accumulate in mitochondria [10]. The lower calcium ion level induces calcium/calmodulin-dependent protein kinase II (CAMKII) expression, resulting in caspase 12 activation [46]. Furthermore, activated caspase 12 could trigger the caspase cascade in response to ER stress. Caspase 9 activation could be achieved by caspase 12 directly or by an Apaf-1/Cyto C mechanism [43, 46]. The activated caspase 9 catalyzes the cleavage of procaspase 3, resulting in apoptosis [43, 46]. In this study, we also found that caspase 12 protein levels declined when rats were treated with H_2_-O_2_ mixture (Figure 3(a)). Therefore, H_2_-O_2_ mixture played an active role in resisting cardiac apoptosis induced by ER stress.

Similar to the injury caused by ischemia-reperfusion, hypoxia and reoxygenation injury caused by CIH is the most important pathophysiological features of OSA [47]. During hypoxia, ATP is decreased, and oxidative phosphorylation of mitochondria is also weakened [48]. When reoxygenation occurs, a large number of oxygen molecules enter mitochondria, and a large number of ROS are generated, including hydroxyl radicals, oxygen radicals, and hydrogen peroxide [48, 49]. Hydroxyl radicals are the most cytotoxic of ROS; H_2_ has a strong ability to eliminate hydroxyl radicals and peroxynitrite [22, 24]. Previous researches have demonstrated 67% H_2_ and 33% O_2_ mixture gas strikingly decreased ROS induced by ischemia-reperfusion in the brain [34, 50], liver [51], and heart [52] in animal models. Clinical studies have reported 67% H_2_ and 33% O_2_ mixture reduced the inspiratory effort in patients with acute severe tracheal stenosis [53] and restored the exhausted supply of CD8+ T cells in patients with advanced colorectal cancer [54]. Our results showed 67% H_2_ and 33% O_2_ mixture gas increased T-SOD and GSH activity and decreased MDA content against the elevated ROS level induced by CIH. Other studies also demonstrated H_2_ could increase catalase activity [33, 55], induce Nrf 2 transcription [56], and elevate heme oxygenase-1 expression [57] against oxidative stress injury.

During hypoxia and reoxygenation, neutrophils are activated, which triggers NADPH oxidase on the cell membrane and induces the production of free radicals [48, 58]. Heymes et al. first reported that NADPH oxidase was expressed in human myocardium [59] and was an important contributor to oxidative stress [60, 61]. In addition, NOX 2 (a subtype of NADPH oxidase) is specifically expressed in the cytomembrane [59] and plays an integral role in the oxidation-reduction signal pathway [62]. Our results revealed that H_2_-O_2_ mixture considerably reduced CIH-induced ROS levels by inhibiting NOX 2 expression (a subtype of NADPH oxidase) (Figure 5). NOX 2 has also been reported to be an inducer of ER stress that mediates apoptosis through a CHOP/CAMKII pathway [63]. Therefore, lower NOX 2 levels suggested decreased ROS levels and CHOP-derived apoptosis when rats were exposed to CIH (Figure 6). Therefore, we considered H_2_-O_2_ mixture to be a safe and effective antioxidant.

## 5. Conclusion

In conclusion, our results revealed that H_2_-O_2_ mixture efficiently improved cardiac dysfunction and structural disorder. The cardioprotective effect of H_2_-O_2_ mixture was due to its ability to decrease ROS levels that were induced by CIH. Furthermore, our results revealed that H_2_-O_2_ mixture dramatically reduced ER stress and apoptosis when rats were exposed to CIH. The data showed evidence that H_2_-O_2_ mixture protected against the cardiac injury induced by CIH.

## Data Availability

The data used to support the findings of this study are included within the article.

## References

[1] Lam C. S., Tipoe G. L., So K. F., Fung M. L. (2015). Neuroprotective mechanism of *Lycium barbarum* polysaccharides against hippocampal-dependent spatial memory deficits in a rat model of obstructive sleep apnea. *PLoS One*.

[2] Young T., Peppard P. E., Gottlieb D. J. (2002). Epidemiology of obstructive sleep apnea: a population health perspective. *American Journal of Respiratory and Critical Care Medicine*.

[3] Brisco M. A., Goldberg L. R. (2010). Sleep apnea in congestive heart failure. *Current Heart Failure Reports*.

[4] Pedrosa R. P., Krieger E. M., Lorenzi-Filho G., Drager L. F. (2011). Recent advances of the impact of obstructive sleep apnea on systemic hypertension. *Arquivos Brasileiros de Cardiologia*.

[5] Liu Y., Yu Z., Hua D., Chen Y., Zheng S., Wang L. (2015). Association of serum hepcidin levels with the presence and severity of obstructive sleep apnea syndrome. *Medical Science Monitor*.

[6] Luo Q., Zhang H. L., Tao X. C., Zhao Z. H., Yang Y. J., Liu Z. H. (2010). Impact of untreated sleep apnea on prognosis of patients with congestive heart failure. *International Journal of Cardiology*.

[7] Huynh N. T., Prilipko O., Kushida C. A., Guilleminault C. (2014). Volumetric brain morphometry changes in patients with obstructive sleep apnea syndrome: effects of CPAP treatment and literature review. *Frontiers in Neurology*.

[8] Sun Y., Yuan H., Zhao M. Q., Wang Y., Xia M., Li Y. Z. (2014). Cardiac structural and functional changes in old elderly patients with obstructive sleep apnoea-hypopnoea syndrome. *The Journal of International Medical Research*.

[9] Gordon P., Sanders M. H. (2005). Sleep.7: positive airway pressure therapy for obstructive sleep apnoea/hypopnoea syndrome. *Thorax*.

[10] Chen R., Huo L., Shi X. (2014). Endoplasmic reticulum stress induced by zinc oxide nanoparticles is an earlier biomarker for nanotoxicological evaluation. *ACS Nano*.

[11] Zhou L., Chen P., Peng Y., Ouyang R. (2016). Role of oxidative stress in the neurocognitive dysfunction of obstructive sleep apnea syndrome. *Oxidative Medicine and Cellular Longevity*.

[12] Xu L. H., Xie H., Shi Z. H. (2015). Critical role of endoplasmic reticulum stress in chronic intermittent hypoxia-induced deficits in synaptic plasticity and long-term memory. *Antioxidants & Redox Signaling*.

[13] Minamino T., Kitakaze M. (2010). ER stress in cardiovascular disease. *Journal of Molecular and Cellular Cardiology*.

[14] Ochoa C. D., Wu R. F., Terada L. S. (2018). ROS signaling and ER stress in cardiovascular disease. *Molecular Aspects of Medicine*.

[15] Yao Y., Lu Q., Hu Z., Yu Y., Chen Q., Wang Q. K. (2017). A non-canonical pathway regulates ER stress signaling and blocks ER stress-induced apoptosis and heart failure. *Nature Communications*.

[16] Aldosari S., Awad M., Harrington E. O., Sellke F., Abid M. (2018). Subcellular reactive oxygen species (ROS) in cardiovascular pathophysiology. *Antioxidants*.

[17] Jomova K., Valko M. (2011). Advances in metal-induced oxidative stress and human disease. *Toxicology*.

[18] Bourdier G., Flore P., Sanchez H., Pepin J. L., Belaidi E., Arnaud C. (2016). High-intensity training reduces intermittent hypoxia-induced ER stress and myocardial infarct size. *American Journal of Physiology-Heart and Circulatory Physiology*.

[19] Cai X. H., Li X. C., Jin S. W. (2014). Endoplasmic reticulum stress plays critical role in brain damage after chronic intermittent hypoxia in growing rats. *Experimental Neurology*.

[20] Ding W., Zhang X., Huang H. (2014). Adiponectin protects rat myocardium against chronic intermittent hypoxia-induced injury via inhibition of endoplasmic reticulum stress. *PLoS One*.

[21] Hou Y., Yang H.'., Cui Z., Tai X., Chu Y., Guo X. (2017). Tauroursodeoxycholic acid attenuates endoplasmic reticulum stress and protects the liver from chronic intermittent hypoxia induced injury. *Experimental and Therapeutic Medicine*.

[22] Ohsawa I., Ishikawa M., Takahashi K. (2007). Hydrogen acts as a therapeutic antioxidant by selectively reducing cytotoxic oxygen radicals. *Nature Medicine*.

[23] Fukuda K., Asoh S., Ishikawa M., Yamamoto Y., Ohsawa I., Ohta S. (2007). Inhalation of hydrogen gas suppresses hepatic injury caused by ischemia/reperfusion through reducing oxidative stress. *Biochemical and Biophysical Research Communications*.

[24] Ohta S. (2011). Recent progress toward hydrogen medicine: potential of molecular hydrogen for preventive and therapeutic applications. *Current Pharmaceutical Design*.

[25] Gao Y., Gui Q., Jin L. (2017). Hydrogen-rich saline attenuates hippocampus endoplasmic reticulum stress after cardiac arrest in rats. *Neuroscience Letters*.

[26] Chen K., Wang N., Diao Y. (2017). Hydrogen-rich saline attenuates brain injury induced by cardiopulmonary bypass and inhibits microvascular endothelial cell apoptosis via the PI3K/Akt/GSK3*β* signaling pathway in rats. *Cellular Physiology and Biochemistry*.

[27] Zhang Y., Xu J., Long Z. (2016). Hydrogen (H_2_) inhibits isoproterenol-induced cardiac hypertrophy via antioxidative pathways. *Frontiers in Pharmacology*.

[28] Yang S. C., Chen L. L., Fu T., Li W. Y., Ji E. S. (2018). Improvement of hydrogen on liver oxidative stress injury in chronic intermittent hypoxia rats. *China Journal of Applied Physiology*.

[29] Zhao Y., Xin Z., Li N. (2018). Nano-liposomes of lycopene reduces ischemic brain damage in rodents by regulating iron metabolism. *Free Radical Biology & Medicine*.

[30] Zhao Y. S., Zhang L. H., Yu P. P. (2018). Ceruloplasmin, a potential therapeutic agent for Alzheimer’s disease. *Antioxidants & Redox Signaling*.

[31] Arias M. A., García-Río F., Alonso-Fernández A., Mediano O., Martínez I., Villamor J́. (2005). Obstructive sleep apnea syndrome affects left ventricular diastolic function: effects of nasal continuous positive airway pressure in men. *Circulation*.

[32] Lai M. C., Lin J. G., Pai P. Y. (2015). Effects of rhodiola crenulata on mice hearts under severe sleep apnea. *BMC Complementary and Alternative Medicine*.

[33] Peng Z., Chen W., Wang L. (2015). Inhalation of hydrogen gas ameliorates glyoxylate-induced calcium oxalate deposition and renal oxidative stress in mice. *International Journal of Clinical and Experimental Pathology*.

[34] Cui J., Chen X., Zhai X. (2016). Inhalation of water electrolysis-derived hydrogen ameliorates cerebral ischemia-reperfusion injury in rats - a possible new hydrogen resource for clinical use. *Neuroscience*.

[35] Fu H. Y., Okada K., Liao Y. (2010). Ablation of C/EBP homologous protein attenuates endoplasmic reticulum-mediated apoptosis and cardiac dysfunction induced by pressure overload. *Circulation*.

[36] Friedman A. D. (1996). GADD153/CHOP, a DNA damage-inducible protein, reduced CAAT/enhancer binding protein activities and increased apoptosis in 32D c13 myeloid cells. *Cancer Research*.

[37] Marciniak S. J., Yun C. Y., Oyadomari S. (2004). CHOP induces death by promoting protein synthesis and oxidation in the stressed endoplasmic reticulum. *Genes & Development*.

[38] Yang X., Shao H., Liu W. (2015). Endoplasmic reticulum stress and oxidative stress are involved in ZnO nanoparticle-induced hepatotoxicity. *Toxicology Letters*.

[39] Zhang P., Sun Q., Zhao C. (2014). HDAC4 protects cells from ER stress induced apoptosis through interaction with ATF4. *Cellular Signalling*.

[40] Szegezdi E., Logue S. E., Gorman A. M., Samali A. (2006). Mediators of endoplasmic reticulum stress-induced apoptosis. *EMBO Reports*.

[41] Ferrandi C., Ballerio R., Gaillard P. (2004). Inhibition of c-Jun N-terminal kinase decreases cardiomyocyte apoptosis and infarct size after myocardial ischemia and reperfusion in anaesthetized rats. *British Journal of Pharmacology*.

[42] Kuwana T., Mackey M. R., Perkins G. (2002). Bid, Bax, and lipids cooperate to form supramolecular openings in the outer mitochondrial membrane. *Cell*.

[43] Morishima N., Nakanishi K., Takenouchi H., Shibata T., Yasuhiko Y. (2002). An endoplasmic reticulum stress-specific caspase cascade in apoptosis. Cytochrome c-independent activation of caspase-9 by caspase-12. *The Journal of Biological Chemistry*.

[44] Urano F., Wang X., Bertolotti A. (2000). Coupling of stress in the ER to activation of JNK protein kinases by transmembrane protein kinase IRE1. *Science*.

[45] McCullough K. D., Martindale J. L., Klotz L. O., Aw T.-Y., Holbrook N. J. (2001). Gadd153 sensitizes cells to endoplasmic reticulum stress by down-regulating Bcl2 and perturbing the cellular redox state. *Molecular and Cellular Biology*.

[46] Xiong Z. G., Zhu X. M., Chu X. P. (2004). Neuroprotection in ischemia: blocking calcium-permeable acid-sensing ion channels. *Cell*.

[47] Gabryelska A., Łukasik Z. M., Makowska J. S., Białasiewicz P. (2018). Obstructive sleep apnea: from intermittent hypoxia to cardiovascular complications via blood platelets. *Frontiers in Neurology*.

[48] Kalogeris T., Baines C. P., Krenz M., Korthuis R. J. (2017). Ischemia/reperfusion. *Comprehensive Physiology*.

[49] Inagaki T., Akiyama T., Du C. K., Zhan D. Y., Yoshimoto M., Shirai M. (2016). Monoamine oxidase-induced hydroxyl radical production and cardiomyocyte injury during myocardial ischemia-reperfusion in rats. *Free Radical Research*.

[50] Huang J. L., Liu W. W., Sun X. J. (2018). Hydrogen inhalation improves mouse neurological outcomes after cerebral ischemia/reperfusion independent of anti-necroptosis. *Medical Gas Research*.

[51] Li H., Chen O., Ye Z. (2017). Inhalation of high concentrations of hydrogen ameliorates liver ischemia/reperfusion injury through A_2A_ receptor mediated PI3K-Akt pathway. *Biochemical Pharmacology*.

[52] Chen O., Cao Z., Li H. (2017). High-concentration hydrogen protects mouse heart against ischemia/reperfusion injury through activation of thePI3K/Akt1 pathway. *Scientific Reports*.

[53] Zhou Z. Q., Zhong C. H., Su Z. Q. (2019). Breathing hydrogen-oxygen mixture decreases inspiratory effort in patients with tracheal stenosis. *Respiration*.

[54] Akagi J., Baba H. (2018). Hydrogen gas restores exhausted CD8+ T cells in patients with advanced colorectal cancer to improve prognosis. *Oncology Reports*.

[55] Liu R., Fang X., Meng C. (2015). Lung inflation with hydrogen during the cold ischemia phase decreases lung graft injury in rats. *Experimental Biology and Medicine*.

[56] Fang W., Wang G., Tang L. (2018). Hydrogen gas inhalation protects against cutaneous ischaemia/reperfusion injury in a mouse model of pressure ulcer. *Journal of Cellular and Molecular Medicine*.

[57] Shen N. Y., Bi J. B., Zhang J. Y. (2017). Hydrogen-rich water protects against inflammatory bowel disease in mice by inhibiting endoplasmic reticulum stress and promoting heme oxygenase-1 expression. *World Journal of Gastroenterology*.

[58] Vandeplassche G., Hermans C., Thoné F., Borgers M. (1989). Mitochondrial hydrogen peroxide generation by NADH-oxidase activity following regional myocardial ischemia in the dog. *Journal of Molecular and Cellular Cardiology*.

[59] Heymes C., Bendall J. K., Ratajczak P. (2003). Increased myocardial NADPH oxidase activity in human heart failure. *Journal of the American College of Cardiology*.

[60] Bedard K., Krause K. H. (2007). The NOX family of ROS-generating NADPH oxidases: physiology and pathophysiology. *Physiological Reviews*.

[61] Guichard C., Pedruzzi E., Fay M. (2006). The Nox/Duox family of ROS-generating NADPH oxidases. *Medecine Sciences*.

[62] Brown D. I., Griendling K. K. (2009). Nox proteins in signal transduction. *Free Radical Biology & Medicine*.

[63] Laurindo F. R. M., Araujo T. L. S., Abrahão T. B. (2014). Nox NADPH oxidases and the endoplasmic reticulum. *Antioxidants & Redox Signaling*.

